# Prevalence and Quality of Endodontic Treatment in Patients with Cardiovascular Disease and Associated Risk Factors

**DOI:** 10.3390/jcm11206046

**Published:** 2022-10-13

**Authors:** Gathani Dash, Lora Mishra, Naomi Ranjan Singh, Rini Behera, Satya Ranjan Misra, Manoj Kumar, Krzysztof Sokolowski, Kunal Agarwal, Suresh Kumar Behera, Sunil Mishra, Barbara Lapinska

**Affiliations:** 1 Department of Conservative Dentistry and Endodontics, Institute of Dental Sciences, Siksha ‘O’ Anusandhan, Bhubaneswar 751003, Odisha, India; 2 Department of Oral Medicine and Radiology, Institute of Dental Sciences, Siksha ‘O’ Anusandhan, Bhubaneswar 751003, Odisha, India; 3 Department of Periodontics, Institute of Dental Sciences, Siksha ‘O’ Anusandhan, Bhubaneswar 751003, Odisha, India; 4 Department of Conservative Dentistry, Medical University of Lodz, 251 Pomorska St, 92-213 Lodz, Poland; 5 Department of Oral Medicine and Radiology, S.C.B. Dental College and Hospital, Cuttack 753001, Odisha, India; 6 Department of Cardiology, Institute of Medical Sciences and SUM Hospital, Siksha ‘O’ Anusandhan, Bhubaneswar 751003, Odisha, India; 7 Department of Cardiology, SUM Ultimate Medicare, Siksha ‘O’ Anusandhan, Bhubaneswar 751003, Odisha, India; 8 Department of General Dentistry, Medical University of Lodz, 251 Pomorska St, 92-213 Lodz, Poland

**Keywords:** cardiovascular disease, caries, endodontics, periapical radiolucency, periodontitis

## Abstract

This study aimed to determine the prevalence and quality of endodontic treatment, by radiographically assessing the periapical periodontitis and endodontic treatment status in patients with cardiovascular disease (CVD) and cardiovascular risk (CVR) factors. Patients who visited the Out Patient Department of Institute of Dental Sciences and Department of Cardiology, Institute of Medical Sciences and SUM Hospital, Siksha ‘O’ Anusandhan University, Bhubaneswar, from August 2021 to February 2022, for a check-up or dental problem were considered as participants in this study. After obtaining informed consent, the participants were enrolled on the Oral Infections and Vascular Disease Epidemiology Study (INVEST) IDS, BHUBANESWAR. After testing negative for COVID-19, patients’ demographic details, such as age and gender were recorded, followed by a panoramic radiographic examination (OPG). A total sample of 408 patients were divided into three groups: Group 1/control (without any cardiovascular manifestation) consisting of 102 samples, group 2 of 222 CVR patients, and group 3 of 84 CVD cases. The CVR and CVD groups had a preponderance of elderly age groups between 60 to 70 years, with a significantly higher proportion of males. Co-morbidities such as diabetes mellitus, hypertension, and dyslipidemia were significantly associated with the CVR and CVD groups. From OPG interpretation, it was observed that the periapical radiolucency was greater in the CVR and CVD groups than in the control group (*p* = 0.009). The prevalence of endodontically treated teeth was higher in CVR and CVD than in the control group (*p* = 0.028). A high prevalence of dental caries, about 70%, was reported in all three groups (*p* = 0.356). The presence of dental restoration among all the groups was low (*p* = 0.079). The proportion of periodontal bone loss in the control group was significantly lower than CVR and CVD (*p* = 0.000). There was a strong association between periapical radiolucency, endodontically treated teeth, and periodontal bone loss in CVR and CVD patients. Notably, the associations reported herein do not reflect a cause-effect relationship; however, individuals with endodontic pathologies may accumulate additional risk factors predisposing them to hypertension or other CVDs. The results emphasize that eliminating local infections may decrease the systemic infection burden.

## 1. Introduction

The prevalence of systemic diseases has risen among the Indian population, without the slightest awareness of their existence [1]. Patients with chronic systemic diseases are frequently associated with oral health problems and disease [2]. These systemic diseases make dental treatment more complicated, along with additional considerations in the dental clinic [2]. Inflammation lasting for prolonged periods is the most common factor associated with systemic and oral diseases, especially in the geriatric population [2]. Dental management becomes more complex in ageing individuals with prolonged periods of medication for systemic diseases, and due to the side effects associated with their medication [2]. As systemic diseases frequently present with oral manifestations, both in soft and hard tissues of the oral cavity, the dental clinician should adopt an appropriate oral health strategy for managing oral diseases [2]. Most common systemic diseases such as diabetes, hypertension, cardiovascular diseases, stroke, and arthritis are frequently associated with xerostomia, dental caries, gingivitis, periodontitis, oral mucosal reactions (angular cheilitis, denture stomatitis, lichenoid reactions, geographic tongue), and fungal infections (candidiasis) [2]. These additional oral health problems further aggravate and deteriorate an individual’s overall health [2].

A demographic study on the Indian population revealed an increasing trend regarding the prevalence of systemic diseases [1]. Cardiovascular disease (CVD) is the leading cause of death worldwide, mainly in low- to middle-income countries [3]. CVD is a heart and vascular system disease and comprises a diverse group of diseases such as coronary heart disease (CHD), peripheral artery disease, and cerebrovascular disease [2]. The most common cardiovascular conditions for patients seeking dental treatment are coronary heart disease, hypertension, dysrhythmia, infective endocarditis, and heart failure [4]. Several studies [3,5,6] have shown an association between poor oral health and coronary heart disease.

Oral health-related quality of life is poor amongst patients with established CVD [3,7]. There have been several studies [3,6,8,9,10] that investigated the association of periodontal disease with CVD. Substantial evidence exists that supports a strong association between periodontal disease and cardiovascular diseases [3,6,9,10,11].

Several systematic reviews and meta-analysis [12,13,14,15,16,17,18,19,20,21] have been conducted, to find out if there is an association between endodontic infections and CVD. However, in these articles the level of evidence about this topic was graded by the respective authors (using AMSTAR 2 tool, GRADE, and ROBINS-I tool) as critically low to moderate. Therefore, a huge research gap has been identified, and further investigation is needed, to explore the association between endodontic lesions and cardiac disease. This study is an attempt to investigate the possibility of an association between lesions of endodontic origin and coronary heart disease.

## 2. Materials and Methods

This study was conducted in the Institute of Dental Sciences, Siksha ‘O’ Anusandhan, Bhubaneswar. Approval for conducting this study was granted by the Institutional Ethics Committee of Institute of Medical Sciences and SUM Hospital, Siksha ‘O’ Anusandhan Deemed to be University, Bhubaneswar. Ethical clearance was granted with I.E.C. Registration Number-ECR/627/Inst/OR/2014/RR-20.

### 2.1. Source of Data

Patients who visited the Out Patient Department (OPD) of Institute of Dental Sciences and Department of Cardiology, Institute of Medical Sciences and SUM Hospital (IMS and SH), Siksha ‘O’ Anusandhan Deemed to be University, Bhubaneswar, from August 2021 to February 2022, for follow-up/check-up or dental problem were considered participants in this study. Informed consent, both in English and Odia language, was obtained from each patient.

All the participants were enrolled under the Oral Infections, and Vascular Disease Epidemiology Study (INVEST) IDS, BHUBANESWAR, and the OPD cards of the eligible participants according to inclusion criteria (Table 1) were stamped as INVEST, IDS, BHUBANESWAR. As the study was carried out during the COVID-19 pandemic period, all those patients who tested COVID-negative were asked for a panoramic radiographic examination, followed by the recording of questionnaire responses. All patients’ findings were coded and recorded in Microsoft Excel Version 16.63.1 (Redmond, WA, USA).

### 2.2. Sample Size Calculation

In this study, the association of oral health-related quality of life associated with endodontic factors in cardiovascular disease patient groups was studied by the Chi-square test of independence. Therefore, sample size determination was performed for the Chi-square test of independence (Algorithm 1.) using G*Power 3.1.9.4 software (Heinrich-Heine University, Düsseldorf, Germany) [22]. Since the prevalence of endodontic factors had two categories and there were three groups, the degree of freedom was 2.
**Algorithm 1.** Formula for calculating sample size**Analysis:** A priori: Compute the required sample size **Input:**  Tail(s)     = Two   Effect size |ρ| = 0.18    α err probability  =  0.05   Power (1 − β err probability)  =  0.95 **Output:**  Noncentrality parameter δ =   3.6183698 Critical t =     1.9660811 Df  =  389 Total sample size  =  39 **Actual power**  =  0.9504731

**Figure 1:**
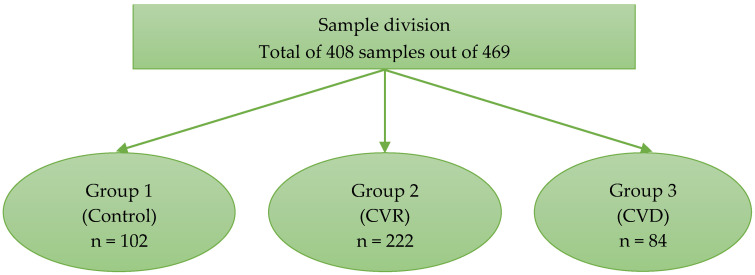
This figure depicts the division of subjects into different groups. Group 1 is the control group or patients with low or borderline cardiovascular risk factor group, Group 2 is the intermediate to high cardiovascular risk factor group (CVR Group), and Group 3 is the established cardiovascular disease group (CVD Group).

The sample size for this study was 408 (102 for control, 222 for CVR, and 84 for the CVD group), which is higher than the minimum sample size of 391 required to achieve a test power of 0.95 for a 0.05 level of significance (Figure 1).

### 2.3. Criteria to Evaluate the Cardiovascular Risk Status (Atherosclerotic Cardiovascular Disease Classification, ASCVD)

The 10-year risk for ASCVD [23] risk for patients aged between 40 and 79 years was categorized into:

1. low-risk (<5%)
2. borderline risk (5% to 7.4%)
3. intermediate risk (7.5% to 19.9%)
4. high risk (≥20%)

### 2.4. CVD Group

CVD is a heart and vascular system disease and comprises a diverse group of diseases, including coronary heart disease (CHD), peripheral artery disease, and cerebrovascular disease [2]. The most common cardiovascular conditions seen among patients seeking dental treatment are coronary heart disease, hypertension, dysrhythmias, infective endocarditis, and heart failure, to name a few [4]. Patients who had undergone angioplasties, CABG procedures, and valve replacement were also placed in this group.

### 2.5. Data Collection

The data collection was carried out by (G.D.). All patients who gave written consent to participate in this study were clinically examined. Information on socio-demographic status was collected from individual participants.

**Figure 2:**
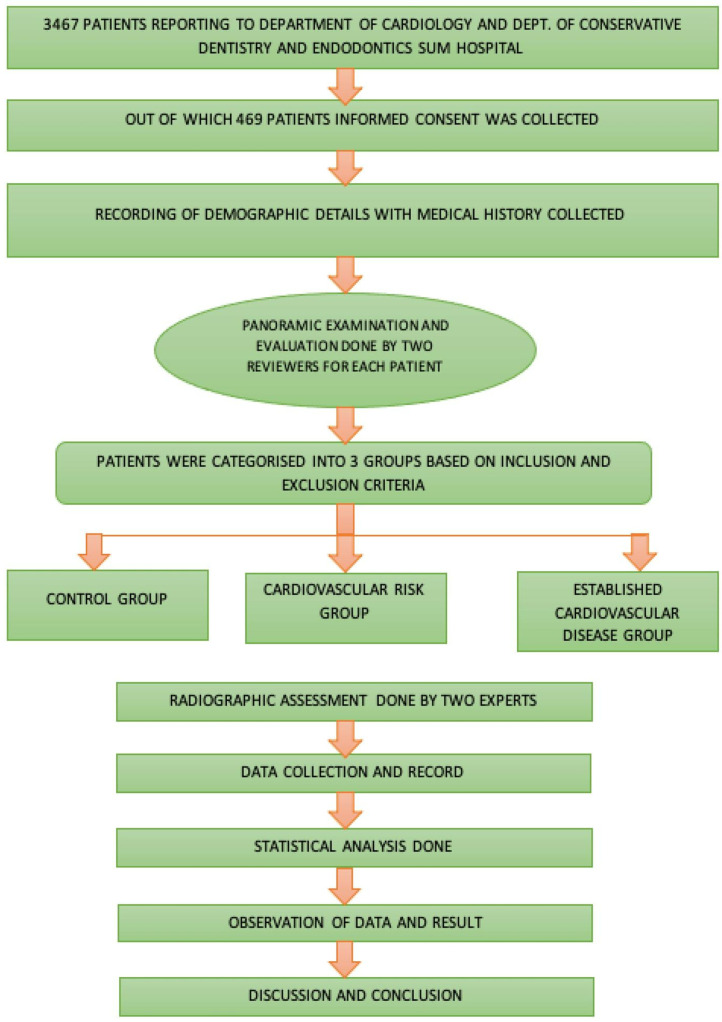
Flowchart of the methodology followed in the present investigation.

Patients who gave consent to be part of the present investigation were asked for a panoramic radiographic (OPG) examination in the Department of Oral Medicine and Radiology, Institute of Dental Sciences, by trained personnel using a CANON OPG MACHINE CU 257 operating at 90 Kv (Carestream Dental L.L.C. Atlanta, GA, USA) (Figure 2).

**Figure 3:**
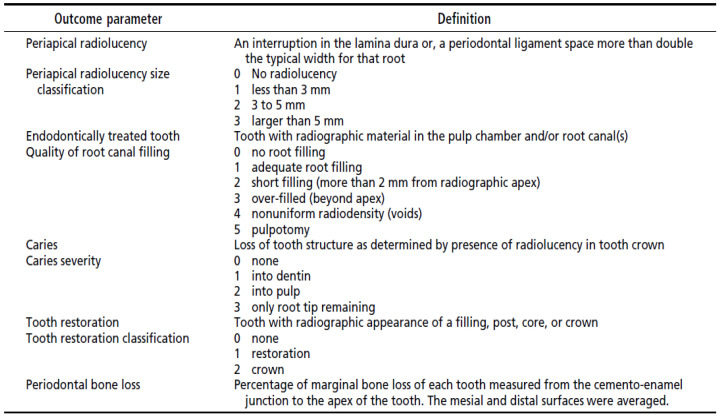
The criteria for the radiographic categorization of teeth.

The obtained panoramic radiographs (OPG) were placed on a viewing box and interpreted by two calibrated expert examiners (N.R.S. and R.B.), to give scores accordingly (Figure 2 and Figure 3). Intra- and inter-examiner reliability was assessed in this study. Intra examiner assessment was done for each evaluator, to calibrate them: the same OPG was shown three times on different days to one examiner, to see if they were consistent with their scoring. As for the inter-examiner evaluation, two reviewers were given same set of OPGs and their scores were recorded, and Cohen kappa assessment was done to check the similarity of the scoring they give for a particular condition.

The radiographic examination included recording the following parameters: caries severity, restorations, periodontal bone loss, endodontically treated teeth, periapical radiolucency, and quality of endodontic treatments. For each parameter recorded from the radiograph, scores were given accordingly (Figure 3).

The severity of alveolar bone loss was represented as a percentage of missing bone at the mesial and distal surfaces of each tooth present and mentioned as teeth with a higher or equal to 50% bone loss, and teeth with a bone loss of less than 50%.

Endodontically treated teeth were examined for the quality of root canal treatment (Figure 3). The quality of the root fillings was assessed by measuring the distance between the root filling termination and the tooth’s radiographic apex. The root fillings were then categorized by the distance from the radiographic apex as follows: less than 2 mm, more than 2 mm, or beyond the radiographic apex. Root filling density was assessed by noting the homogeneity of fill and whether voids were present. The presence and size of periapical radiolucency were categorized as none, less than 3 mm in diameter, 3 to 5 mm in diameter, and larger than 5 mm. For multirooted teeth, the largest radiolucency was recorded. After the endodontic consultation, endodontic information was recorded in the patient’s clinical record.

Information on five key endodontic factors was obtained:

1. number of teeth requiring endodontic treatment,
2. tooth type,
3. whether the tooth/teeth previously had root canal treatment,
4. diagnosis based on Ingle’s classification of pulp and periapical status [24] (If a patient had multiple teeth requiring endodontic treatment, the diagnostic classification of the tooth about which the patient chiefly complained was considered),
5. periapical radiolucency was based on the methods and criteria of the peri-apical index (PAI) from periapical radiographs [25]. The highest score amongst the teeth was assigned to the participant; for multirooted teeth, the highest score of any individual root was assigned.

### 2.6. Statistical Analysis

Data collected under the study were scrutinized, coded, and entered into IBM SPSS Statistics, 24.0 software (www.spss.co.in, Armonk, NY, USA, accessed on 10 May 2022), for analysis. The following statistical procedure was used for the analysis of data.

Association of categorical variables such as age group, gender, diabetes mellitus (DM), hypertension (HTN), dyslipidemia, smoking, oral health status, periapical radiolucency, endodontically treated tooth, caries severity, tooth restoration, and periodontal bone loss were evaluated using a cross-tabulation procedure. Their associations were studied using the Chi-square test of independence. Agreement on periapical radiolucency, endodontically treated tooth, caries severity, tooth restoration, and periodontal bone loss between examiners 1 and 2 was quantified using the Kappa test. A value of *p* less than 0.05 was considered to indicate the cut-off for statistical significance.

## 3. Results

Data were collected from 408 patients. Among these 408 patients, 102 were in group 1 (control), 222 in group 2 (CVR), and 84 in group 3 (CVD).

### 3.1. Demographic and Clinical Profile of Cases

#### 3.1.1. Age and Gender Distribution

There were 33.1% patients in the ≤50 years, 30.9% in 50–60 years, 27.5% in 61–70 years, and 8.6% in the >70 years age group. The proportion of patients ≤50 years in the control group was significantly higher than in groups 2 and 3 (*p* = 0.000). This implied cases with cardio-vascular manifestation were from a more elderly population. Males and females were 61.3% and 38.7%, respectively. The proportion of males was significantly higher in groups 2 and 3 compared to group 1 (*p* = 0.010). Table 2 presents the demographic details.

#### 3.1.2. Clinical Profile of Cases

Table 3 presents the association of the clinical profile of cases with groups. Of 408 cases, 245 patients (60%) had no DM, whereas 163 (40%) had DM. The proportion of DM was the highest in the CVR group (57.7%), followed by CVD (41.7%) and nil in the control group (*p* = 0.000). There was also a significant DM difference between groups 2 and 3 (*p* = 0.012).

The proportion of HTN cases in the CVD group was significantly higher, i.e., 57.7%, than in groups 1 and 2 (*p* = 0.000). However, there was no significant difference in HTN proportion between groups 2 and 3 (*p* = 0.069).

Similarly, a significant difference in dyslipidemia proportion among the three groups was observed (*p* = 0.000). The dyslipidemia proportion was the highest among CVD (46.4%), followed by group 2 (CVR) (2.3%) and nil in the control. The proportion of smoking in group 2 was significantly higher, i.e., 14% than in group 1 (Nil) and group 3 (9.5%) (*p* = 0.000). Between groups 2 and 3, no significant difference in the proportion of smoking was observed (*p* = 0.299).

Table 4 depicts the frequency distribution of ASCVD risk factors among CVR cases. The majority of cases had an intermediate risk (38.7%), followed by nearly one third of high-risk cases. Nearly 20% had a low to borderline risk.

### 3.2. Agreement on OPG Interpretation between Examiners

The overall agreement between the two calibrated examiners (N.R.S. and R.B.) was 84.4%. The Kappa value showed an excellent agreement between examiners 1 and 2 (Kappa = 0.781) with (*p* = 0.000).

### 3.3. Endodontic and Periodontal Status among the Investigated Population Sample

**Figure 4:**
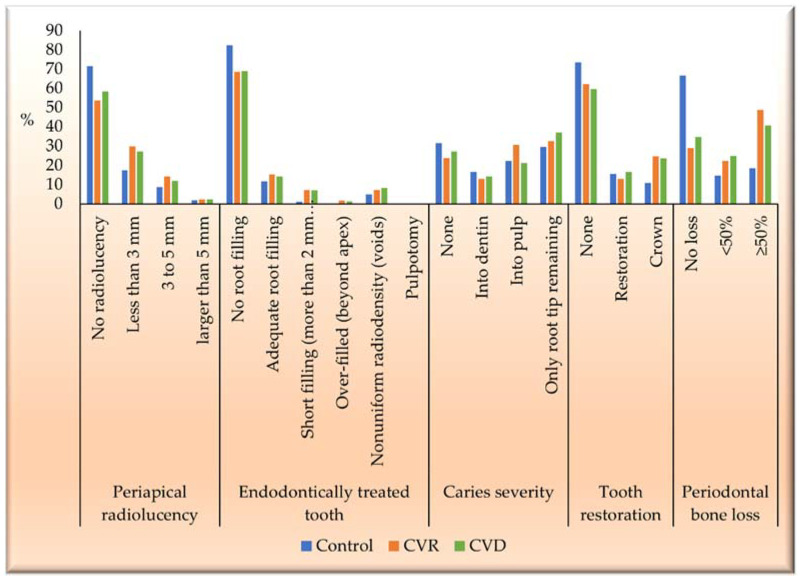
The overall score for each parameter assessed using OPG.

The prevalence of endodontic and periodontal manifestations by group is presented in Table 5 and Figure 4. Out of 408 cases, 167 (40.9%) had radiolucency. The prevalence of radiolucency was significantly lower in group 1 (28.4%) than in group 2 (46.4%) and 3 (41.7%), (*p* = 0.009). However, between groups 2 and 3, there was no significant association of prevalence (*p* = 0.458).

In endodontically treated teeth, 114 (27.9%) had a root canal filling, of which 18 (17.6%) belonged to the control, 70 (31.5%) had CVR, and 26 (31%) had CVD. The proportion of root canal filling in group 1 was significantly lower than in groups 2 and 3 (*p* = 0.028). However, between groups 2 and 3, there was no significant association of prevalence (*p* = 0.922).

Regarding caries severity, 108 (26.5%) cases had no caries, and 300 (73.5%) had caries. The proportion of caries severity cases was evenly distributed among the groups. There was no significant difference among and between groups 2 and 3 (*p* > 0.05).

As for tooth restorations, 263 (64.5%) cases had no tooth restoration, and 145 (35.5%) had restoration. The proportion of tooth restoration cases was evenly distributed among the three groups. There was no significant difference between the three groups and between groups 2 and 3 (*p* > 0.05).

When assessing periodontal bone loss, in 247 (60.5%) cases, bone loss was detected, whereas 161 (39.5%) cases had no bone loss. Out of the bone loss cases, 34 (33.3%) were in the control, 158 (71.2%) in the CVR group, and 55 (65.5%) in the CVD group. It can be seen that the proportion of periodontal bone loss was significantly lower in group 1 than in groups 2 and 3 (*p* = 0.000). Nevertheless, between groups 2 and 3, the proportion of periodontal bone loss was evenly distributed, and the association was insignificant (*p* = 0.934).

## 4. Discussion

In this study, OPG was used as an examination tool for investigating the prevalence of some common oral diseases of the hard tissues in the tested population. Panoramic radiographs are helpful in community studies for diagnosing oral diseases [26]. Despite having limitations, such as a lower accuracy in the detection of periapical radiolucency and failed root canal treatments than a complete intraoral radiographic series [27,28], they have several advantages. Involving less time than the intraoral full mouth radiographs with less radiation exposure, requiring less patient cooperation, and having a broader scope of diagnostic possibilities have led to an increased use of OPGs in several extensive population studies [26,29,30]. It was reported that studies that used a periapical radiograph resulted in a higher prevalence of people with at least one tooth with AP (56%) than studies that used OPG (46%). However, a combination of both image methods yielded a proportion similar to the periapical radiograph alone (60%) [31]. The present study was conducted at the peak of the COVID-19 pandemic, hence OPG was an excellent tool to investigate oral health without inserting instruments into the patient’s oral cavity.

Interpretation of a large number of radiographs usually needs to be done by multiple observers [26]. The reading of dental radiographs may involve interobserver and intra-observer variances, and previous studies have shown that the best agreement is achieved if there are two observers [32,33]. In the current study, two examiners were trained and calibrated, and strict criteria for recordings were set up before the start of the examination.

### 4.1. Demographic Characteristics of the Sample

Our study observed that the proportion of males was significantly higher in CVR and CVD groups. These findings follow other studies, which also evaluated a preponderance of the elderly age group. As age advances, there may be a higher risk of cardiovascular manifestations, with the proportion of males being significantly higher than females [26,34]. The possible reason for the lower CVD prevalence among females is due to the endogenous estrogens during the fertility period in women, delaying the manifestation of atherosclerotic disease [35]. Estrogens regulate several metabolic factors, such as lipids, inflammatory markers, and the coagulant system, and promote a direct vasodilatory effect through the α and β receptors in the vessel wall [35]. Due to estrogen hormones in females, until the menopause period, they are at lower risk than males [35]. In addition, it has been seen that men are less adaptive to coping with stressful conditions, therefore, predisposing themselves to an increased risk for CAD [36].

### 4.2. Clinical Profile of the Sample

The clinical profile of the cases for both CVR and CVD groups was assessed in patients who reported DM, HTN, dyslipidemia, and smoking. DM was prevalent in both the CVR (57.7%) and CVD groups (41.7%). Another study investigating oral health issues among CVD patients observed that patients with diabetes were 1.4-times more prone to problems than the non-DM group [37]. The possible reason for the poor oral health among these patients could be that the salivary *Porphyromonas gingivalis* was higher in the uncontrolled DM group than in well-controlled DM subjects. The CVD with DM cases further escalated the numbers of these microorganisms [38]. The authors of the same study also suggested that the presence of specific periodontopathic bacterial infection might be the factor that aggravates DM in CVD patients [38]. *Porphyromonas gingivalis* is a Gram-negative, anaerobic bacteria detected in 85% of chronic periodontal infections and a component of subgingival microbiomes that promotes dysbiosis of oral flora [39]. Its prevalence in diabetic and CVD patients can trigger endothelial dysfunction by activating endothelial cells and platelets, recruitment of leukocytes, and the proliferation and migration of smooth muscle cells (SMCs), leading to the formation of a lipid core, thus aggravating atherosclerosis [39]. The presence of virulence factors such as lipopolysaccharides (LPS), heat shock protein-60 (HSP-60), fimbria, outer membrane vesicles (OMVs), and enzymes such as gingipains can trigger proinflammatory conditions, along with foam cell formation and vascular calcification, enhancing oxidative stress and exerting deteriorative effects on the healing process of the infarcted myocardium, and subsequently leading to cardiac rupture and AMI [39]. Periodontal treatment can improve endothelial function, reducing the biomarkers (such as CRP, IL-6, TNF-α, fibrinogen, and triglycerides) of atherosclerotic disease, particularly in individuals already suffering from CVDs [39].

Another risk factor associated with CVD and CVR patients is HTN. In our study, the proportion of HTN cases in the CVD group was significantly higher, at 57.7%, compared to the control and CVR groups. In CVR and CVD, poor oral hygiene, rampant dental caries, and periapical diseases are observed. These oral conditions predispose these patients to the risk of cardiovascular events [40,41,42]. Oral hygiene maintenance is imperative for these patients [41,42]. Our finding follows another similar investigation, which concluded that periapical abscesses were significantly higher in hypertensive patients, with more prevalence in patients with secondary hypertension than with primary hypertension [41]. The possible reason for this could be the hyposalivation seen in hypertensive patients. HTN patients are often on antihypertensive medications, especially diuretics, to maintain systolic and diastolic blood pressure [43]. These medications decrease the unstimulated saliva, resulting in a higher prevalence of dental caries in these patients [43]. As disturbances of calcium metabolism are seen in hypertensive patients under medication with calcium channel blockers, e.g., nifedipine, a decrease in bone density is seen, which leads to a delayed response of bone to noxious stimuli, such as inflammation, along with the bone healing capacity from infection, resulting in a higher prevalence of periodontal abscesses [41].

Another risk factor that predisposes patients to cardiovascular disease is increased cholesterol levels. Cholesterol imbalance, otherwise known as dyslipidemia, is a pathognomonic sign among CVD and CVR patients. This imbalance is most likely to predispose an individual to periodontal disease and vice versa. The reason for this could be long-standing chronic periodontal infections. These infections can trigger local or systemic inflammatory conditions, due to bacterial products and their endotoxins (LPS) when released into the bloodstream, stimulating the production of cytokines such as tumor necrosis factor-α (TNF-α) and interleukin-1β (IL-1β), and subsequently leading to endothelial dysfunction and atherogenesis, especially in elderly patients above 65 years of age with an increased risk of cardiac disease [44,45,46,47]. Therefore, healthcare professionals should suggest CVR and CVD patients have periodic dental check-ups and educate them on maintaining adequate oral health and hygiene [46].

It is well-known that smoking also increases the risk of developing cardiovascular diseases among patients [46,48]. The chemicals in cigarette smoke cause the cells that line blood vessels to become swollen and inflamed. These byproducts of smoking can narrow the blood vessels and lead to many cardiovascular conditions [46,48]. Smoking affects the general well-being of a person and negatively impacts individuals’ oral health. Several studies have concluded that smoking supports the growth of cariogenic bacteria, plaque, and tartar build-up, leading to cavities, tooth decay, and tooth loss, as well as mouth sores and ulcers in the oral cavity, thus harming oral health status [46,48,49,50,51,52,53,54,55,56]. The possible reasons for such oral findings could be that smoking alters the oral microbiome by increasing the acidity of saliva, impairing blood flow to the gums, and depleting oxygen, which helps bacterial adherence to the mucosal surfaces, causing decreased host immunity [51]. Gram-positive to Gram-negative bacteria [51] are prevalent in developing plaques in smokers, favoring the survival of facultative anaerobes over strict aerobes, especially *Streptococcus* [51]. The authors in another investigation evaluated the effect of smoking on oral health and concluded that smoking cessation improved cardiovascular outcomes and oral health [48].

### 4.3. Prevalence of Periapical Radiolucency in the Investigated Sample

In the present investigation, we observed that the prevalence of periapical radiolucency was higher in the CVR (46.4%) and CVD (41.4%) groups when compared to the control group (28.4%). Periapical radiolucency is the radiographic sign of periapical inflammation. The primary source of these lesions is a microbiological, chemical, or mechanical trauma to the pulp, which causes necrosis or inflammation and, if left untreated, progresses into bony lesions limited to the apical area of the tooth [57]. Chronic inflammation in the periapical tissues usually develops without any specific clinical symptoms. Therefore, radiographic images are still essential [57].

Some studies have shown the interaction of cytokines resulting from apical periodontitis (AP) lesions, with pro-inflammatory and immunoregulatory mechanisms [58,59]. A persistent inflammatory condition can influence the cardiovascular system, leading to CVD [58,59]. Our findings were similar to another study that concluded that CVDs were more common in patients with AP [60]. These results imply that persistent infection in the periapical region causes a higher oral infection burden among these patients [60].

### 4.4. Quality of Endodontic Treatment in the Investigated Sample

The prevalence of endodontically treated teeth was higher in CVR (31.5%) and CVD (31%) compared to the control group (17.6%). The success of endodontic therapy depends on an appropriately performed root canal therapy (RCT), including adequate root canal filling and post-endodontic restoration. Inadequate obturation, poor quality root canal filling and unfinished RCT can perpetuate a dead space for bacterial growth. Only 5% of the sample had an inadequate root canal filling in the control group. In the CVR group, 16.2% of cases had an inadequate root canal filling. Similarly, 16.6% of cases had an inadequately filled root canals in the CVD group. This result implies that the quality of endodontic treatment provided to the patients was poor, and the need for re-treatment is high.

An investigation of the impact of unfinished root canal treatment on CVR concluded that a high number of unfinished RCTs were independently associated with a high risk of future CVD hospitalization [61]. The reason for this could be that the root canal flora of teeth with an intact clinical crown and necrotic pulp is dominated by obligate aerobes, including *Fusobacterium*, *Porphyromonas*, *Prevotella*, *Eubacterium,* and *Peptostreptococcus* [62]. In case of an incomplete or unfinished root canal therapy, these microorganisms can indirectly elevate inflammatory mediator levels and cytokine concentration, such as interleukins (IL-6, IL-2) [63,64]. This low-grade chronic inflammation may be a risk factor for the development of atherosclerosis and CVD, because of its ability to induce endothelial dysfunction [65].

### 4.5. Prevalence and Severity of Dental Caries

In our study, all groups had a high prevalence of dental caries. In the control group, 68.6% reported dental caries, with 76.1% in the CVR group and 72.6% in the CVD group. The steep rise in the prevalence of dental caries among all groups could have been due to the COVID-19 pandemic. In this period, the number of dental treatment seekers was dramatically reduced during the initial lockdown period [66]. It has generally been observed that oral healthcare is often ignored due to the burden of other health-related duties and the priority given to medical care. Another reason for the increased prevalence of dental caries could be poor access to oral health care services or an inability to afford the endodontic treatment provided by nearby private clinics [26].

Several studies evaluated the prevalence or severity of dental caries as a risk factor for CVD [8,41,42,67,68,69,70,71]. The authors concluded that caries, especially in the advanced/severe stage, were significantly associated with increased CHD risk.

### 4.6. Prevalence of Tooth Restorations

The presence of dental restoration among all the groups was low; with 73.5% in the control group, 62.2% of CVD, and 59.5% of CVR having no restorations. The proportion of tooth restoration among the three groups did not have a significant association. This result implies that although the investigated sample had a high prevalence of dental caries and treatment requirements, a significantly smaller number of the population received dental restoration. This could be due to a lack of knowledge and awareness of the dental problems among the population investigated.

### 4.7. Prevalence of Periodontal Bone Loss

The evaluation of the periodontal bone loss among the investigated population revealed that the proportion of periodontal bone loss in the control group (33.3%) was significantly lower when compared to the CVR (71.7%) and CVD (65.5%) groups. The OPG also revealed that the periodontal bone loss was ≥50% in the CVR and CVD groups, and the findings were significant compared to the control group. Our findings are in accordance with several other studies [6,8,72,73,74,75,76]. The possible reason for the prevalence of periodontal bone loss in CVR and CVD patients is the increase in inflammatory mediators/cytokines and CRP for a prolonged period, which causes progressive destruction of connective tissue attachment and an increase in the alveolar bone loss evident radiographically, leading to pocket formation [76]. This can subsequently lead to tooth mobility, a decrease in masticatory function, and eventual tooth loss [6,76]. Both endodontic and periodontal diseases have a great influence on endodontic treatment outcomes [72,73]. The presence of chronic endodontic infection can progress to periodontitis, leading to deeper periodontal pockets, advanced radiographic attachment loss, and angular bone defects [73]. In one investigation, the authors demonstrated an approximately three-fold increase in marginal proximal radiographic bone loss by endodontic infections in periodontitis-prone patients, with an average loss of 0.19 mm/year, compared to 0.06 mm/year without endodontic infection [73].

Periodontitis is also a non-communicable disease, with a high prevalence of 45%–50% overall, with the most severe form affecting 11.2% of the world’s population, and being the sixth most common human disease [10,77]. Severe periodontitis has been independently and significantly associated with all-cause and cardiovascular mortality in several populations [78,79].

There is evidence of significantly higher levels of C-reactive protein (CRP) and elevated levels of serum interleukin (IL-6) in periodontitis patients and lower levels of IL-4 and IL-18 in periodontitis patients versus healthy controls and CVD and periodontitis patients compared with either condition alone [64,79]. Peripheral neutrophils from periodontitis patients release excess IL-1β, IL-8, IL-6, and tumor necrosis factor (TNF-α) when stimulated by periodontal pathogens [77]. Periodontal therapy only partially reduces cytokine hyper-reactivity, with some evidence of a constitutively elevated response [79].

## 5. Limitations of the Study

Primarily due to the COVID-19 pandemic, a small number of patients were examined, which is the main limitation of this study. In addition, among the recruited patients, the control group was found to be was significantly younger than the CVR and CVD groups. Hence, it could be expected that the prevalence of dental issues, including endodontic treatment and periapical infection, would be higher in those groups. However, the population selected was 40 years and above, and the reason for selecting this age group was that cardiac diseases are most prevalent in the 40–80 years age group. Dental decay is a multifactorial disease and age cannot be the only primary determinant of dental disease. Moreover, it was reported that healthy individuals had a lower frequency of teeth with apical periodontitis than individuals with systemic conditions, such as e.g., cardiovascular disease [31]. Second, we used panoramic radiographs to score the endodontic and periodontal lesions, which is a two-dimensional representation, and the actual size of the lesion could not be assessed. Lastly, in terms of biological effects, taking histological samples might be better for assessing the severity of lesions.

## 6. Conclusions

Both cardiologists and general physicians should advise patients to maintain good oral health and educate them about the negative impact of poor oral health status on overall health.

Within the limitations of this study, we can conclude that the cardiac risk and cardiac disease populations had a poor oral health status. In our study, there was a strong association between periapical radiolucency, endodontically treated teeth, and periodontal bone loss in CVR and CVD patients. Notably, the associations reported herein do not reflect a cause-effect relationship; however, individuals with endodontic pathologies may accumulate additional risk factors predisposing them to hypertension and other CVDs. The results emphasize that eliminating local infections may decrease the systemic infection burden. Patients should be asked to maintain effective periodontal therapy, as it positively affects the reduction of cardiovascular events.

## Figures and Tables

**Table 1:** Inclusion and exclusion criteria of patients selected under the INSPIRE scheme.

Inclusion Criteria	Exclusion Criteria
Patients who visited OPD of Institute of Dental Sciences and Department of Cardiology, IMS and SUM Hospital,	Patients with severe medical conditions and who were hospitalized
Patients between 40–80 years of age.	Patients with physical disabilities (mobility, visual, or hearing impairments)
Patients with primary dental experience or who required dental intervention	Any communication difficulties were noted on the patient’s record
	Pregnant patients

**Table 2:** Age and gender distribution of the investigated sample.

Age Group	Group 1 (Control)		Group 2 (CVR)		Group 3 (CVD)		Total		χ^2^, *p*
	*n*	%	*n*	%	*n*	%	*n*	%	
**≤50**	62	60.8	52	23.4	21	25	135	33.1	χ^2^ = 56.927 *p* = 0.000
**51-60**	27	26.5	70	31.5	29	34.5	126	30.9	
**61-70**	11	10.8	79	35.6	22	26.2	112	27.5	
**>70**	2	2	21	9.5	12	14.3	35	8.6	
**Gender**									
**Male**	50	49	148	66.7	52	61.9	250	61.3	χ^2^ = 9.190 *p* = 0.010
**Female**	52	51	74	33.3	32	38.1	158	38.7	
**Total**	102	100	222	100	84	100	408	100	

**Table 3:** Clinical profile of the investigated sample.

DM	Group 1 (Control)		Group 2 (CVR)		Group 3 (CVD)		Total		χ^2^, *p*
	*n*	%	*n*	%	*n*	%	*n*	%	
**No**	102	100	94	42.3	49	58.3	245	60	χ^2^ = 96.977 *p* = 0.000
**Yes**	0	0	128	57.7	35	41.7	163	40	
**χ^2^, *p*-value for association of DM with Gr. 2 and 3: χ^2^ = 6.260, *p* = 0.012**									
**HTN**									
**No**	102	100	94	42.3	26	31	222	54.4	χ^2^ = 117.133 *p* = 0.000
**Yes**	0	0	128	57.7	58	69	186	45.6	
**χ^2^, *p*-value for association of HTN with Gr. 2 and 3: χ^2^ = 3.317, *p* = 0.069**									
**Dyslipidemia**									
**No**	102	100	217	97.7	45	53.6	364	89.2	χ^2^ = 140.050 *p* = 0.000
**Yes**	0	0	5	2.3	39	46.4	44	10.8	
**χ^2^, *p*-value for association of dyslipidemia with Gr. 2 and 3: χ^2^ = 96.600, *p* = 0.000**									
**Smoking**									
**No**	102	100	191	86	76	90.5	369	90.4	χ^2^ = 15.764 *p* = 0.000
**Yes**	0	0	31	14	8	9.5	39	9.6	
**χ^2^, *p*-value for association of smoking with Gr. 2 and 3: χ^2^ = 1.080, *p* = 0.299**									
**Total**	102	100	222	100	84	100	408	100	

**Table 4:** Frequency distribution of ASCVD risk factors among CVR cases.

ASCVD Risk Factor	*n*	%
Low-risk (<5%)	42	18.9
Borderline risk (5% to 7.4%)	23	10.4
Intermediate risk (7.5% to 19.9%)	86	38.7
High risk (≥20%)	71	32
Total	222	100

**Table 5:** Prevalence of endodontic and periodontal manifestations in the investigated sample.

	Group 1 (Control)		Group 2 (CVR)		Group 3 (CVD)		Total		χ^2^, *p*
	*n*	%	*n*	%	*n*	%	*n*	%	
**Periapical radiolucency**									
**No radiolucency**	73	71.6	119	53.6	49	58.3	241	59.1	χ^2^ = 9.353 *p* = 0.009
**Radiolucency**	29	28.4	103	46.4	35	41.7	167	40.9	
**χ^2^, *p*-value for Gr. 2 and 3: χ^2^ = 0.009, *p* = 0.922**									
**Endodontically treated tooth**									
**No root canal filling**	84	82.4	152	68.5	58	69	294	72.1	χ^2^ = 7.168 *p*= 0.028
**Root canal filling**	18	17.6	70	31.5	26	31	114	27.9	
**χ^2^, *p*-value for Gr. 2 and 3: χ^2^ = 0.009, *p* = 0.922**									
**Caries severity**									
**None**	32	31.4	53	23.9	23	27.4	108	26.5	χ^2^ = 2.064 *p*= 0.356
**Severity**	70	68.6	169	76.1	61	72.6	300	73.5	
**χ^2^, *p*-value for Gr. 2 and 3: χ^2^ = 0.402, *p* = 0.526**									
**Tooth restoration**									
**None**	75	73.5	138	62.2	50	59.5	263	64.5	χ^2^ = 5.067 *p* = 0.079
**Restoration**	27	26.5	84	37.8	34	40.5	145	35.5	
**χ^2^, *p*-value for Gr. 2 and 3: χ^2^ = 0.179, *p* = 0.672**									
**Periodontal bone loss**									
**No loss**	68	66.7	64	28.8	29	34.5	161	39.5	χ^2^ = 42.964 *p* = 0.000
**Loss**	34	33.3	158	71.2	55	65.5	247	60.5	
**χ^2^, *p*-value for Gr. 2 and 3: χ^2^ = 0.934, *p* = 0.334**									
**Total**	102	100	222	100	84	100	408	100	

## Data Availability

Not applicable.
